# Optimization of mass spectrometry settings for steroidomic analysis in young and old killifish

**DOI:** 10.1007/s00216-020-02640-6

**Published:** 2020-04-24

**Authors:** Rahel Dabrowski, Roberto Ripa, Christian Latza, Andrea Annibal, Adam Antebi

**Affiliations:** 1 Max Planck Institute for Biology of Ageing, Joseph-Stelzmann-Strasse 9 b, 50931 Cologne, Germany; 2 Cologne Excellence Cluster on Cellular Stress Responses in Aging-Associated Diseases (CECAD), University of Cologne, Josef-Stelzmann-Strasse 26, 50931 Cologne, Germany

**Keywords:** Mass spectrometry, Orbitrap, Killifish, Steroidomics, Ageing

## Abstract

Steroids are essential structural components of cell membranes that organize lipid rafts and modulate membrane fluidity. They can also act as signalling molecules that work through nuclear and G protein–coupled receptors to impact health and disease. Notably, changes in steroid levels have been implicated in metabolic, cardiovascular and neurodegenerative diseases, but how alterations in the steroid pool affect ageing is less well understood. One of the major challenges in steroidomic analysis is the ability to simultaneously detect and distinguish various steroids due to low in vivo concentrations and naturally occurring stereoisomers. Here, we established such a method to study the mass spectrometry behaviour of nine sterols/steroids and related molecules (cholesterol precursors: squalene, lanosterol; sterol metabolites; 7 Dehydrocholesterol, 24, 25 and 27 Hydroxycholesterol; and steroids: progesterone, testosterone, and corticosterone) during ageing in the African turquoise killifish, a new model for studying vertebrate longevity. We find that levels of all tested steroids change significantly with age in multiple tissues, suggesting that specific steroids could be used as biomarkers of ageing. These findings pave the way for use of *Nothobranchius furzeri* as a novel model organism to unravel the role of sterols/steroids in ageing and age-related diseases.

**Electronic supplementary material:**

The online version of this article (10.1007/s00216-020-02640-6) contains supplementary material, which is available to authorized users.

**Figure:**
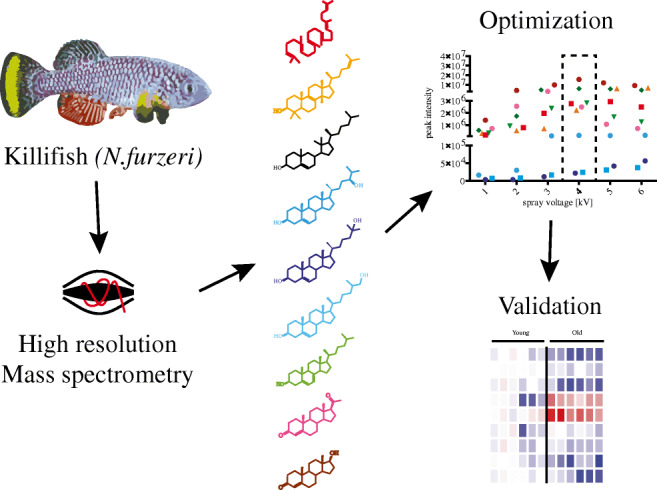
Graphical abstract

## Introduction

Sterols and steroids are essential heterocyclic molecules used by all metazoans for diverse biological processes. They are important structural components of cell membranes that comprise lipid rafts and decrease membrane fluidity [1]. They also act as signalling molecules that typically work through nuclear and G protein–coupled receptors to regulate metabolism, development, and homeostasis, [2] and their dysregulation can lead to disease.

The most abundant sterol is cholesterol, which is salvaged from the diet or de novo synthesized through a series of steps. A critical step in cholesterol synthesis is the cyclization of squalene to lanosterol, which can then be converted into cholesterol through multiple parallel pathways, one of which is via 7 Dehydrocholesterol [3]. Subsequently, cholesterol can undergo several modifications and transmutation reactions leading to a plethora of sterol/steroid-like molecules with different biological functions including oxysterols, bile acids, steroids, corticoids, and secosteroids [4].

Dysregulation of cholesterol synthesis and steroid levels in different tissues are associated with oxidative stress and cell death, which can contribute to age-related diseases and pathological conditions [5, 6]. For example, increased levels of oxysterols have been implicated in Alzheimer’s disease (AD), atherosclerosis, type 2 diabetes, and cancer [7–9]. In addition, in mice and humans blood steroid concentration changes with age, [10–12] and longitudinal studies have identified a correlation between age-related decline in steroid hormones and metabolic syndrome (MetS) as well as cardiovascular disease (CVD) [13, 14].

Given the importance of various steroids in physiology and age-related disease it is essential to clarify their role in vivo*,* and dissect how their abundance and regulation changes with age. However, such efforts are currently hampered by the fact that vertebrate steroids are found at very low concentrations in vivo varying from 0.5 to 50 pg/mL in different tissues [15, 16], making it difficult to obtain enough material for extraction. Second, steroids often exist as stereoisomers and methods are lacking to separate and identify these distinct moieties. Third, analysing steroid levels in longitudinal studies remains challenging in humans. To overcome these problems, we established a model system using the African turquoise killifish and high-resolution mass accuracy mass spectrometry, which enables the detection of steroids in vivo and also allows us to reliably investigate their role in ageing. The African turquoise killifish *Nothobranchius furzeri* (*N. furzeri*) is an emerging model for the study of ageing and age-related diseases [17, 18]. *N. furzeri* lives only 6 to 7 months, and is among the shortest-lived vertebrates that can be kept in laboratory conditions [19]. They display many features of human ageing, including loss of mobility, cancer, and cognitive decline [20, 21]. Notably, in killifish steroidogenesis occurs primarily in gonads, adrenal glands, liver, and brain [22–24]. Fish also typically contain high concentrations of steroids, facilitating the study of steroids in vivo [25–27].

Another important consideration is the ability to measure different biologically relevant steroid species in parallel. Traditionally, gas chromatography-mass spectrometry (GC-MS) has been used for oxysterol analysis in cells, tissues, and plasma [28, 29]. GC-MS methods provide high information content, but require extensive sample preparation, making it a laborious option for routine or repeated analyses. As an alternative, electrospray ionization (ESI) coupled to liquid chromatography-mass spectrometry (LC-MS) is the preferred method for high-throughput studies and reverse-phase LC-MS has been extensively employed for the analysis of estradiol, testosterone, and corticosterone in human urine, plasma and serum [30–32]. However, none of the currently described methods are able to detect more than six steroids of interest with high sensitivity [15, 33].

In the current study, we thus established a highly sensitive high-resolution mass spectrometry-based method to measure nine different steroids in the killifish *N. furzeri* during the ageing process.

## Material and methods

### Chemicals

UHPLC-grade water, UHPLC-grade acetonitrile, and formic acid were purchased from Biosolve Valkenswaard, Netherlands. UHPLC methanol and ethyl acetate were purchased from Sigma-Aldrich, GmbH. Isopropanol was purchased from Carl Roth GmbH und Co. KG (Karlsruhe, Germany) and Chloroform was purchased from Merck KGaA (Darmstadt, Germany). The following standards were obtained from Sigma-Aldrich, GmbH: 7 Dehydrocholesterol, 24(S) Hydroxycholesterol, 25 Hydroxycholesterol, 27 Hydroxycholesterol, corticosterone, lanosterol, progesterone, squalene, 7-Ketocholesterol (d7), and testosterone as well as tricaine methanesulfonate.

### LC-MS instrumentation

LC-MS analysis was carried out on a Q-Exactive Plus, (Thermo Fischer Scientic GmbH, Bremen, Germany). Steroid standards were separated using a XSelect column (HSS T3 2,5 μm 2.1 × 100, Waters) and a binary pump system (Vanquish, ThermoFisher Scientific GmbH, Bremen, Germany) with solvent A as water with 0.1% v/v formic acid and eluent B as acetonitrile with 0.1% v/v formic acid. The gradient started with 10% eluent B and kept for 0.3 min., then ramped to 99% eluent B in 8 min and held for 2 min. in 1 min it decreased to 10% eluent B and kept it for 1 min. The total time for the gradient was 12 min and the column was kept at 45 °C.

To narrow down the crucial settings to maximize the sensitivity for the quantification of steroid in killifish samples, we explored several mass spectrometry parameters using steroid standards (100 ng). Spray voltage was set from 1 to 6 kV in positive ion mode. Then, mass resolving power 17.500, 30.000, 70.000, 140.000, and 280.000 were compared and automated gain control (AGC) settings 2e^4^, 5e^4^, 1e^5^, 2e^5^, 5e^5^, 1e^6^, 3e^6^, and 5e^6^ were tested. Lastly, injection time (IT) 100, 150, 200, and 250 ms were examined, while sheet gas and auxiliary gas were kept constant at 20 and 5 respectively as well as S-lenses at 60 (a.u.).

We then examine the following liquid chromatography parameters for optimal steroid identification: flow rates 50, 100, 150, and 200 μL/min as well as needle positions A, B, C, and D from the mass spectrometer inlet.

During the analysis we monitored the following ions: squalene- > 411.3985, lanosterol- > 427.3934, 7 Dehydrocholesterol- > 385.3465, 24–25- and 27 Hydroxyholesterol- > 403.3571, progesterone- > 315.2319, corticosterone- > 347.2217, testosterone- > 289.2162.

Data was analysed using Xcalibur version 4.0 and Trace Finder version 4.1.

### Killifish husbandry and tissue collection

All experiments were performed using the *N. furzeri* short-lived strain GRZ-AD. Following fertilization, eggs were collected by sieving the sand with a plastic net and kept in wet peat moss during embryonic development. Embryos were then hatched by flushing the peat with tap water at 16–18 °C and transferred to a clean vessel with a cut plastic pipette. Fry were fed with newly hatched *Artemia nauplii* for the first 2 weeks and then weaned with finely chopped *Chironomus* larvae. Subsequently, fish were raised individually in 2.8-L tanks at 27 °C and fed two times a day with frozen *Chironomus* larvae or living nauplii of *Artemia salina*. For tissue collection, fish were sacrificed using an overdose of tricaine methanesulfonate (TMS, MS-222) (0.4 mg/mL) and brain, liver, gonads and gut tissue was immediately extracted by dissection. Tissues were frozen in liquid nitrogen and stored at − 80 C°. For ageing analysis, we collected samples from six 5-week-old and six 15-week-old wild-type male fish.

### Steroids extractions from killifish tissues

Tissues underwent up to three thaw and freeze cycle in liquid nitrogen and were then homogenized by bead beatings for 30 min at 50 oscillations/s at 4 °C using the Qiagen tissue lyser. A volume corresponding to 100 μg proteins was subjected to three different extraction methods, namely Bligh and Dye, methanol extraction, and solid-phase extraction (SPE). Before extraction, 2 ng of internal standard (7-Ketocholesterol-d7) was added. In the first method, 150 μL chloroform/methanol 2:1 v/v was added to the samples, whereas in the second method, 100 μL of cold methanol was added. In both methods, samples were afterwards rotated for 1 h at 4 °C and centrifuged for 10 min at 10.000 rpm. The supernatant was transferred to a new tube in both methods and dried using a speedvac as described in our previous works [34, 35]. The third method used SPE to enrich the sterol fraction: HLB prime cartridges (Oasis, Waters) were preconditioned with 1 mL methanol and 1 mL water with 1% *v*/v formic acid before adding the sample. After the sample was applied, phospholipids were removed by adding 1 mL hexane. Steroids were eluted using 500 μl ethyl acetate as previously described [36]. The organic fraction was dried in a speed vac as described above. Twenty microliters of 50% v/v methanol/water was used to reconstitute the sample and 5 μL was injected into the LC-MS/MS system. The relative response for each steroid species was calculated by dividing the peak area of the analyte to the internal standard peak area (7-Ketocholesterol-d7) and further normalized to protein concentration.

### Statistics

Statistical analyses were performed using GraphPad Prism software 7.03. *P* values were calculated with *t* test.

## Results

### Optimization of mass spectrometry settings

**Scheme 1:**
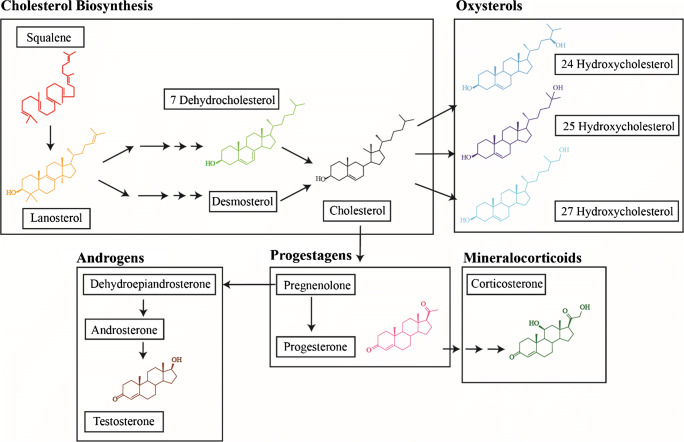
Chemical structures of the main steroids. Steroids are shown in groups (Cholesterol Biosynthesis, Oxysterols, Progestagens, Mineralcorticoids and Androgens) and in order of synthesis. Colours of chemical structures are identical with colours of data points in the result figures

We used ultra-high pressure liquid chromatography (UHPLC) coupled with electrospray mass spectrometry (ESI-MS) for the detection and identification of nine steroids (Scheme 1). Since steroids are present at low concentrations in vivo we first optimized different parameters to increase sensitivity. In all optimizations, we report the mean intensity for each standard in the figures, whereas the corresponding relative standard deviations (RSD) are reported in Electronic Supplementary Material (ESM) Tables (S1-S7). The measurements have a RSD ranging from 0.28% to 12–13%.

The separation gradient was kept constant in all optimization experiments. Squalene eluted at 2.25 min, lanosterol at 8.03 min, 7 Dehydrocholesterol at 9.83 min, progesterone at 7.48 min, corticosterone at 5.93 min, testosterone at 6.53 min (Table 1). Although the three oxysterols have the same *m/z* value, they eluted at different times: 24 Hydroxycholesterol at 9.84 min, 25 Hydroxycholesterol at 8.91, and 27 Hydroxycholesterol at 10.2 min.

**Table 1:** ESI-MS/MS linear parameters for steroids

Compound	m/z	*t*_R_ (min)	LOD (pg/mL)	LOQ (pg/mL)	Slope	Intercept	*R*^2^
Squalene	411.3985	2.25	0.51	1.71	3.01E+04	1.89E+06	0.94
Lanosterol	427.3934	8.03	0.32	1.06	2.57E+06	1.79E+04	0.99
7 Dehydrocholesterol	385.3465	9.83	0.19	0.62	4.99E+05	9.86E+05	0.90
24 Hydroxyholesterol	403.3571	9.84	1.00	9.79	1.83E+05	2.74E+06	0.94
25 Hydroxycholesterol	403.3571	8.91	2.63	3.57	4.07E+05	1.74E+06	0.99
27 Hydroxyholesterol	403.3571	10.2	3.59	4.56	5.47E+05	8.11E+05	0.99
Progesterone	315.2319	7.48	2.94	3.34	4.98E+05	4.57E+06	0.91
Corticosterone	347.2217	5.93	1.07	8.76	4.94E+05	4.57E+06	0.99
Testosterone	289.2162	6.53	1.37	11.96	3.78E+05	1.77E+06	0.91

**Fig. 1:**
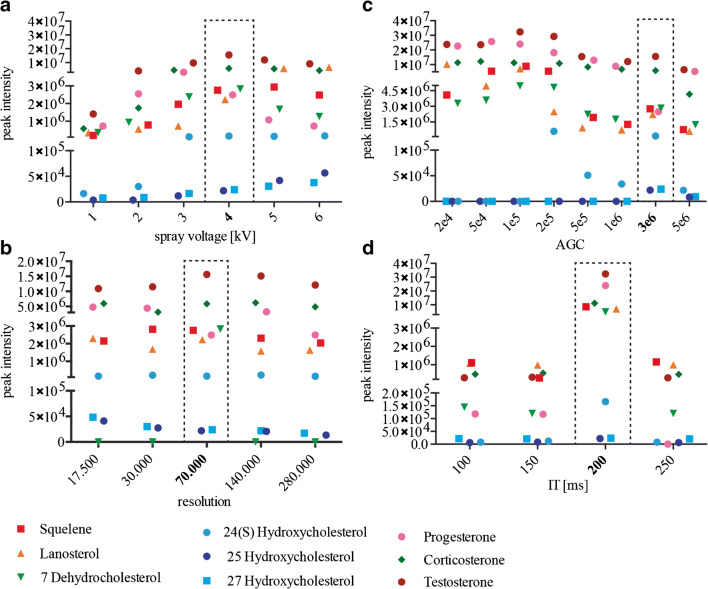
Optimization of the MS parameters: MS parameters that were tested in this study. **a** Peak intensities of all tested compounds with different spray voltages. **b** Peak intensities of all tested compounds with all available resolutions. **c** Peak intensities of all tested compounds with several AGC. **d** Injection times. Each data point is the mean value of an experimental triplicate. Relative standard deviations for all measurements are reported in ESM Tables S1-S4. Optimal conditions are indicated using a dotted box

We initially investigated the effect of the spray voltage on nine standards (Fig. 1a). All nine species were detected from 1 up to 6 kV.

Ion intensity was shown to be proportional to the magnitude of the voltage. An increase of voltage leads to a stronger electric field and by consequence, a stronger force pulls the ion spray into the mass spectrometer. Oxysterols generally followed this expected pattern and while peak intensities were generally low, they increased with higher voltages. Similarly, lanosterol showed higher peak intensity at higher voltage. The other steroids/sterols (squalene, corticosterone, testosterone, progesterone and 7 Dehydrocholesterol, 24 Hydroxycholesterol) did not show this pattern and peak intensity either plateaued or decreased at higher spray voltage. However, higher voltages may also cause in-source fragmentation. When 5 kV and 6 kV were used, the intensity of the ions decreases in comparison with 4 kV. We observed highest peak intensities for most species at a spray voltage of 4 kV and thus performed all further optimizations with this voltage.

We next explored all available resolutions ranging from 17.500 to 280.000 (Fig. 1b). In general, high resolution is required for complex samples and in particular for the separation of stereoisomers. Nevertheless, high resolution can also lead to a decrease in sensitivity because it requires longer Orbitrap scan times. Peak intensities of the oxysterols, corticosterone and lanosterol did not differ greatly between the tested resolutions. Resolutions 17.500 and 70.000 resulted in slightly higher peaks for corticosterone and lanosterol. Testosterone peak intensity was also higher at 70.000 resolution and 7 Dehydrocholesterol could only be detected with a resolution of 70.000. We therefore continued the optimization using the resolution at 70.000.

In terms of ion population, we selected optimal automatic gain control (AGC) target values at 2e^4^, 5e^4^, 1e^5^, 2e^5^, 5e^5^, 1e^6^, 3e^6^, and 5e^6^ (Fig. 1c). AGC controls the number of ions that will enter the Orbitrap. A higher AGC will cause space charge effects and decrease sensitivity. In addition, the scan rate is slowed as the AGC increases, which further affects the sensitivity of the measurement. Most steroids showed highest or second highest peak intensities with an AGC of 1e^5^. However, oxysterols could only be detected with AGCs 2e^5^ or higher. 25 and 27 Hydroxycholesterol could even only be detected with an AGC of 3e^6^. Consequently, we continued the parameter optimization using an AGC of 3e^6^.

We analysed peak intensities of the steroid standards at varying injection times (IT) while the resolution is kept constant at 70.000 (Fig. 1d). The IT can dramatically affect the sensitivity of the analysis, since it determines how long the ions can accumulate in the C-trap. Peak intensities for most steroids were by far the highest with an injection time of 200 ms, whereas higher injection time (250 ms) decreased the intensities of all standards. We thus proceeded with 200 ms.

**Fig. 2:**
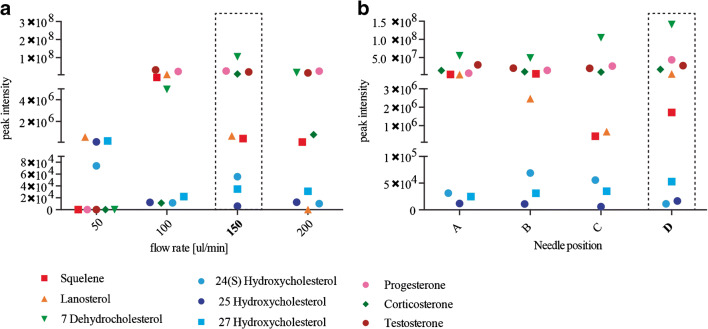
Optimization of the ion source and liquid chromatography parameters: LC parameters that were tested in this study. **a** Peak intensities of all tested compounds with different flow rates. **b** Peak intensities of all tested compounds with needle positions A, B, C, or D. Each data point is the mean value of an experimental triplicate. Relative standard deviations for all measurements are reported in ESM Tables S5-S6. Optimal conditions are indicated using a dotted box

In our attempt to further optimize the MS settings, we tested two parameters of the liquid chromatography: flow rate and needle position (Fig. 2 a and b). The decrease of the mobile phase rate also decreases the droplet radius, which generally turns in an increase in sensitivity. However, we found that 50 μL/min (the slowest flow rate we tested) resulted in the lowest peak intensities. Peak intensities of oxysterols as well as squalene and 7 Dehydrocholesterol were highest with a flow rate of 150 μL/min. Based on our results, we decided to carry on our optimization using a flow rate of 150 μL/min.

Finally, we analysed changes in sensitivity by varying needle positions A, B, C, and D (Fig. 2b), which changes the distance between the electrospray needle and the MS inlet. With the exception of squalene, lanosterol and 24 Hydroxycholesterol position D, which results in the shortest distance between the needle and the inlet, provided the highest peak intensities and thus produced the best results for steroid identification overall.

## Method validation

Using the optimizations described above we established an LC-MS method for steroid analysis with the following parameter settings: 4 kV spray voltage, resolution of 70.000, 3e^6^ AGC, 200 ms IT, 150 μL/min flow rate and needle position D. These settings were validated using serial dilutions of the nine standards from 0.001 nmol/L to 500 nmol/L (Tables 1 and 2). Intraday and interday variability were calculated for all steroid standards. RSD for both measurements were below 15% of variation. Standards were also spiked in killifish matrix prior or after SPE enrichment to calculate recovery rate. Detection limits (LOD) ranged from 0.19 pg/mL for 7 Dehydrocholesterol to 2.94 pg/mL for progesterone. Moreover, quantification limits (LOQ) ranged from 0.62 to 11.96 pg/mL, which are two to three-fold improved compared to previous works (ESM Table S10) [30, 37, 38]. All external calibration curves exhibited high levels of linearity (> 0.90). The measurement using Orbitrap allowed obtaining the elemental composition of each steroid using less than 5 ppm mass accuracy (ESM Table S8).

**Table 2:** Intraday, interday error and recovery of the LC-MS method

Compound	Intraday precision (*n* = 5)	Interday precision (*n* = 5)	Recovery (%)
	ng ± SD (%RSD)	ng ± SD (%RSD)	
7-Ketocholesterol (d7)	1.938 ± 0.16 (8.29)	1.85 ± 0.17 (9.15)	81.73
Squalene	0.544 ± 0.07 (12.97)	1.106 ± 0.083 (7.52)	91.93
Lanosterol	1.049 ± 1.08 (11.97)	2.366 ± 0.30 (13.03)	88.61
7 Dehydrocholesterol	0.780 ± 0.05 (6.80)	2.011 ± 0.28 (14.01)	94.18
24 Hydroxyholesterol	1.268 ± 0.06 (5.37)	1.105 ± 0.09 (8.27)	84.92
25 Hydroxycholesterol	1.077 ± 0.05 (5.12)	1.75 ± 0.20 (11.68)	86.25
27 Hydroxyholesterol	1.267 ± 0.15 (11.99)	0.584 ± 0.071 (12.15)	78.53
Progesterone	0.465 ± 0.46 (2.29)	1.217 ± 0.12 (10.31)	73.82
Corticosterone	1.097 ± 1.16 (10.7)	0.816 ± 0.083 (10.26)	93.81
Testosterone	0.915 ± 0.12 (13.59)	1.961 ± 0.15 (7.83)	81.00

### Steroid extraction and in vivo quantification of steroids in killifish

**Fig. 3:**
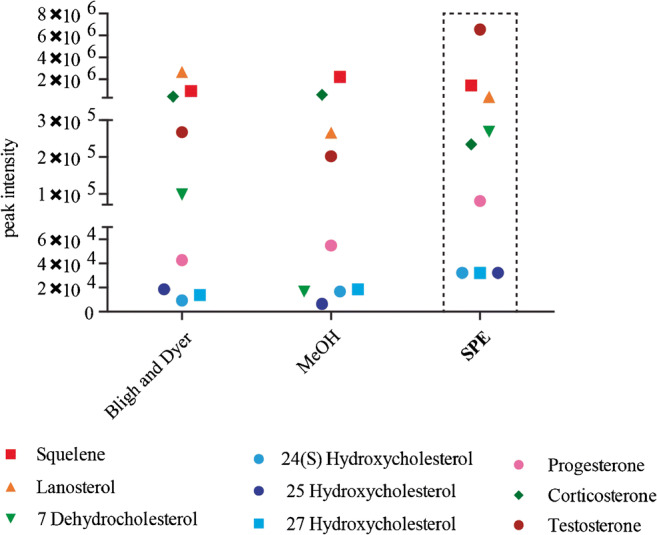
Evaluation of three different methods for steroid extraction from killifish tissues. Peak intensities of all tested compounds using Bligh and Dyer, MeOH extraction or SPE. Each data point is the mean value of an experimental triplicate. Relative standard deviations for all measurements are reported in ESM Table S7. Optimal conditions are indicated using a dotted box

After we established optimal LC-MS parameters for the detection of steroids, we next used the newly developed method to investigate the concentration of selected steroids in killifish tissues. For this we first optimized steroid extractions from tissue using wild-type killifish gonads. We analysed peak intensities of all nine steroids (Fig. 3). This showed that with the exception of lanosterol and corticosterone, the intensity for most species was highest using the SPE enrichment. SPE removes salts and other contaminants that can potentially influence the sensitivity of the measurement. Thus, we used SPE in combination with our new LC-MS method to extract and identify steroids from four different tissues, namely gut, liver, brain, and gonad in young (5-week-old ) and (15-week-old) killifish.

**Fig. 4:**
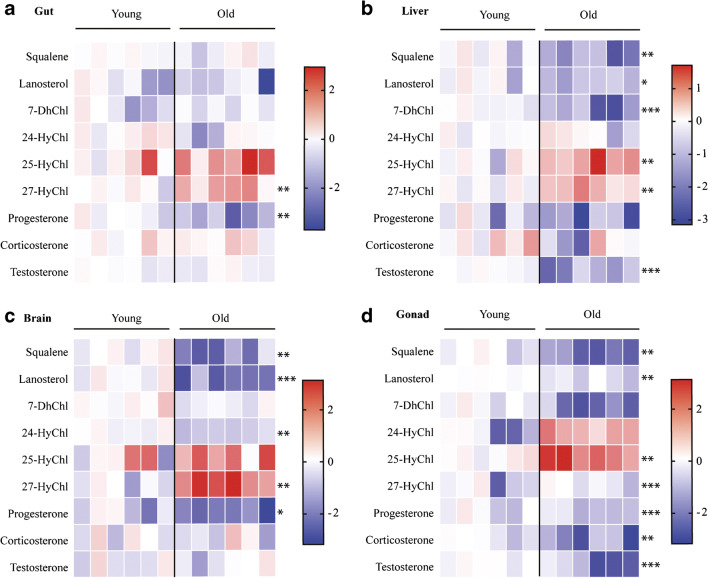
In vivo concentrations of steroids in young and old killifish in the gut, liver, brain, and gonad. Heat map of steroid concentrations from gut (**a**), liver (**b**), brain (**c**), and gonads (**d**) of six young and six old male fish. Steroid concentrations are depicted on a log10 scale. High concentrations are coloured red and low concentrations are coloured blue. Each column represents a single individual. Original data are reported as histograms in the ESM Figs. S1-S4. Statistical tests were performed using *t* test. * *P* ≤ 0.05, ** *P* ≤ 0.01, *** *P* ≤ 0.001

Our results are represented as heatmaps for all tissues (Fig. 4 a–d) and histograms for individual steroids in each tissue are included in the ESM (Figs. S1-S4).

Overall, we found that the level of almost all steroids change with age, with the most change occurring in the gonad and the least change observed in the gut. 25 and 27 Hydroxycholesterol showed an increase in all tissues with age (with statistically significant increases in the liver and gonads), while all other steroids were unchanged or detected at lower quantities in aged tissues. Squalene and lanosterol concentrations were significantly reduced in old fish compared to young in all tested tissues apart from the gut, ranging between two-fold to four-fold of reduction (Fig. 4 b–d). 7 Dehydrocholesterol concentrations showed a tendency to be reduced in aged fish, but the reduction was only statistically significant in the liver (Fig. 4b). Quantification of 24 hydroxycholesterol levels showed an approximately two-fold reduction in the brain of 15-week-old fish compared to 5-week-old fish but their levels remained unchanged in gonads and the liver (Fig. 4b, d). Levels of progesterone and testosterone significantly decreased in gonads during ageing (Fig. 4d). In particular, progesterone levels were also reduced in other tissues (more than three-fold in the brain and about two-fold in the gut) in aged tissues (Fig. 4a, c), while testosterone and corticosterone levels showed less change in brain and gut tissues. However, they decreased significantly in the liver and gonads.

## Discussion

In this study we developed a method for the detection of nine steroids from different tissues of the African turquoise killifish, *N. furzeri*, using LC-MS. The analysis of steroids remains a challenge due to their low concentrations in vivo and their structural similarities [39]. Our newly developed method, however, allows a sensitive detection and simultaneous quantification of nine steroids from different classes.

We optimized both technical parameters of the mass spectrometry and tissue extraction.

We observed a different mass spectrometric behaviour of the steroid standards while we were optimizing each parameter. When using higher voltages at the electrospray source needle, it is likely that discharge can occur between the needle and the counter electrode corona above certain potentials. This results in unstable spray and thereby decreases the signal intensity. However, this was observed only for few steroids, whereas the other steroids were less affected. Lubin A, and Leinonen A, already described this discrepancy, which is associated with the intrinsic physical-chemical properties of the compound and the presence of different function groups, such as 1,4-diene-3-one structure, hydroxyl groups, or carbonyl groups, which affects proton affinity and by consequences ionization efficiency of the different steroids [40, 41].

We also observed the differential behaviours of the steroids at different resolutions. The resolution is dependent on the scan time of the Orbitrap. The higher the resolution, the longer the time the Orbitrap needs to separate the ions, which may affect sensitivity (about 512 ms for resolution of 140,000). Nevertheless, this effect is not visible under our conditions. As described by Kalli A et al., a possible explanation could be, that the concentration of the standard mix is too high for the effect on the resolution to be seen [42], in particular at resolution 280.000. In this optimization, we used sub-molar concentrations of the standards, 100 ng, which is not very far from the physiological concentration (ranging from 0.5 pg/mL to 5 ng/mL in brain and liver) and allowed an easy detection and identification of the steroid species (ESM Table S8).

The AGC target values also dramatically influence the sensitivity for the detection of the steroid standards. Knarchenko et al. investigated these effects using a simulation approach [43]. High AGC target values will result in a slower scan rate and possibly space charge effects, which negatively affect the sensitivity and mass accuracy. On the contrary, lower AGC values lead to less accumulation of ions in the C-trap, which results in lower sensitivity.

We show for the first time a different behaviour of steroid ions at different AGC values.

The reason why the oxysterols could be detected only from AGC values above 2e^5^ remains unclear. We can speculate that either the hydrophobicity of the compounds might have an effect during ion accumulation, or as described by Szabó PT et al., the hydrophobicity of the molecules can affect the ionization process and reduce the number of ions, which can further enter the mass spectrometer [44].

We also report for the first time the effect of injection time on the detection of steroid species using a constant resolution and AGC value. These three parameters are linked together and optimization needs to be done to assess which combination allows higher signal intensity for the desired molecules. As described previously by Michalski A et al., when the injection time is reached, the ions will be injected into the Orbitrap, regardless of whether the AGC target is reached [45]. We showed that the combination of AGC target 3e6 and 200 ms of injection time is beneficial for steroid detection and identification. Based on the concentration of steroid species we can speculate that most of the ions are accumulated in the C-trap and reached the desired AGC values in a time frame that is very close to the selected injection time.

Steroids generally represent a small fraction of the total lipidome of cells and the organism and multiple analytical techniques to extract and enrich for steroids have been reported [29, 46]. Our comparison of three extraction methodologies is in good agreement with previously published data. We show that solid-phase extraction (SPE) remains the golden standard for the purification and enrichment of most species. We additionally include a hexane wash to remove the hydrophobic lipids as suggested by Azadmard-Damirchi S. et al., which resulted in higher intensity and increased sensitivity of the sterol-like molecules compared to other extraction methods [47].

To our knowledge, this study is the first report of the parallel quantification of nine steroids directly from killifish tissues of different ages. The comparison between young and old killifish indicates a dysregulation of steroid levels across multiple tissues with age [10, 48, 49]. We observed a reduction in the levels of intermediates of the cholesterol biosynthesis pathway such as squalene, lanosterol, and 7 Deyhdrocholesterol. These findings are in line with reports that the activity of the sterol regulatory element-binding protein (SREBP) transcription factors, which activate the transcription of enzymes involved in the cholesterol biosynthesis pathway, decrease due to reduced mTORC1 activity during ageing [50–52]. Unfortunately, due to the extraction method and ionization techniques used we were not able to measure free cholesterol concurrently with other steroids as it requires different ionization and instrument set up [53].

Oxysterols are proposed biomarkers of several pathophysiological conditions, such as obesity, atherosclerosis and are involved in immune and inflammatory processes, bone marrow homeostasis and nervous system development [54–56]. Consistently, their levels change during ageing as they impinge on various essential cellular pathways. In line with previous experiments, we show that oxysterol levels are altered during ageing. It has been reported that 24 Hydroxycholesterol levels are decreased in Huntington’s disease (HD) and late AD patients [57]. The reduction in 24 Hydroxycholesterol levels in the brain of aged fish, which we show here, is expected since 24 Hydroxycholesterol levels in the brain are a marker for the metabolic activity of neurons and also the number of neurons, both of which are known to decrease with age [9].

In contrast, it has been shown that levels of 25 and 27 Hydroxycholesterol increase during ageing, particularly in the eye lenses of aged humans, causing cataracts. Additionally, 27 Hydroxycholesterol levels are elevated in atherosclerosis patients and breast tumour cells [58]. The 27 Hydroxycholesterol accumulation in gonad and liver could result from an age-related reduction in CYP27A1 levels, which is the enzyme responsible for its biosynthesis [59].

Ageing is well known to be accompanied by decreasing levels of sex steroid hormones. Our results further support this finding, as both progesterone and testosterone decrease with age in killifish. In men and in rats, a decrease of testosterone levels during ageing is associated with decreasing supply of steroid precursors by Leydig cells [60]. This age-related decline in testosterone steroidogenesis is consistent with decreased mitochondrial expression of steroidogenic acute regulatory protein (StAR) and steroidogenic CYP450s (e.g.CYP11A1) in old versus young rats [61]. We additionally identify estradiol in few tissues, though they were far below the limit of quantification as we used a cohort of only male fish [62].

Taken together, these findings suggest that sex hormones and oxysterols can be developed as biomarkers of ageing and perhaps age-related disease. Further studies will shed light on steroidogenesis and sex hormone production in the newly established model killifish and will reveal new potential endocrine regulators of ageing and ageing-related processes.

## Conclusion

In the current study we thus established a highly sensitive mass spectrometry-based method as a novel tool to study the role of steroids during the ageing process. Using the one-variable-at-time strategy is a viable approach to investigate potential interactions between mass spectrometry parameters. These findings emphasize the importance of thorough parameter optimization during method development.

In general, it is of great importance to find biomarkers of ageing to monitor pathology and age-related diseases. In this regard, it will be interesting in the future to evaluate the possible correlation of steroid concentrations in killifish and ageing. We are confident that this method can be potentially used to investigate sterols in different model organisms but ultimately to improve the steroid detection and quantification in human tissues.

Furthermore, it would be important to assess how genetically or pharmacologically altered steroid quantities alter animal longevity in order to truly understand their role in ageing.

## Electronic supplementary material

ESM 1 (PDF 745 kb)
