# Supplementary material for: Optimization of mass spectrometry settings for steroidomic analysis in young and old killifish
Source: Anal Bioanal Chem. 2020 Apr 24;412(17):4089–99. doi: 10.1007/s00216-020-02640-6 (PMC7320053; doi:10.1007/s00216-020-02640-6)
Supplement: Supplementary file 1 — (PDF 745 kb) [file 216_2020_2640_MOESM1_ESM.pdf]

Analytical and Bioanalytical Chemistry

Electronic Supplementary Material

Optimization of mass spectrometry settings for steroidomic analysis in young and old killifish

Rahel Dabrowski, Roberto Ripa, Christian Latza, Andrea Annibal, Adam Antebi

Table S1 Optimization of spray voltage

Spray voltage / Compound	1 kV		2 kV		3 kV		4 kV		5 kV		6 kV	
	mean	RSD (%)	mean	RSD (%)	mean	RSD (%)	mean	RSD (%)	mean	RSD (%)	mean	RSD (%)
Squalene	2.02E+05	4.37	7.89E+05	6.91	1.96E+06	6.69	2.76E+06	7.30	2.94E+06	4.39	2.48E+06	5.77
Lanosterol	3.42E+05	2.68	5.54E+05	4.43	7.23E+05	1.92	2.23E+06	8.63	5.73E+06	2.12	6.63E+06	9.04
7 Dehydrocholesterol	3.60E+05	3.41	9.41E+05	4.35	2.38E+06	10.98	2.83E+06	12.58	1.68E+06	5.65	1.27E+06	5.79
24 Hydroxycholesterol	1.62E+04	5.57	3.02E+04	8.67	1.20E+05	2.44	1.66E+05	11.73	1.50E+05	2.27	1.74E+05	5.97
25 Hydroxycholesterol	3.80E+03	4.29	3.62E+03	6.68	1.20E+04	4.96	2.20E+04	9.68	4.19E+04	5.32	5.67E+04	7.48
27 Hydroxycholesterol	7.67E+03	1.92	8.61E+03	5.48	1.65E+04	6.06	2.39E+04	5.39	3.07E+04	11.74	3.78E+04	4.50
Progesterone	7.28E+05	9.16	2.55E+06	9.44	3.08E+06	10.86	2.49E+06	13.67	1.08E+06	10.68	7.25E+05	5.66
Corticosterone	5.83E+05	5.33	1.75E+06	7.86	4.54E+06	8.77	5.85E+06	4.21	5.55E+06	1.06	4.38E+06	3.32
Testosterone	1.41E+06	10.77	4.01E+06	5.75	9.78E+06	3.56	1.56E+07	8.52	1.20E+07	4.10	9.18E+06	10.69

Table S2 Optimization of resolution

Resolution / Compound	17,500		30,000		70,000		140,000		280,000	
	mean	RSD (%)	mean	RSD (%)	mean	RSD (%)	mean	RSD (%)	mean	RSD (%)
Squalene	2.15E+06	8.77	2.82E+06	3.25	2.76E+06	7.30	2.31E+06	1.39	2.04E+06	9.80
Lanosterol	2.30E+06	6.63	1.69E+06	9.49	2.23E+06	8.63	1.58E+06	12.80	1.63E+06	5.08
7 Dehydrocholesterol	0.00E+00	0.00	0.00E+00	7.74	2.83E+06	12.58	0.00E+00	0.00	0.00E+00	0.00
24 Hydroxycholesterol	1.68E+05	7.21	2.19E+05	5.88	1.66E+05	11.73	2.20E+05	11.10	1.58E+05	7.72
25 Hydroxycholesterol	4.10E+04	15.63	2.76E+04	7.99	2.20E+04	9.68	2.07E+04	9.81	1.35E+04	9.01
27 Hydroxycholesterol	4.84E+04	8.34	3.02E+04	5.84	2.39E+04	5.39	2.20E+04	1.75	1.72E+04	7.14
Progesterone	4.75E+06	6.25	4.41E+06	10.97	2.49E+06	13.67	3.20E+06	9.62	2.49E+06	0.28
Corticosterone	5.97E+06	5.20	3.10E+06	3.88	5.85E+06	4.21	6.20E+06	1.74	4.86E+06	5.10
Testosterone	1.09E+07	1.06	1.15E+07	1.82	1.56E+07	8.52	1.51E+07	7.37	1.21E+07	2.34

Table S3-1 AGC Parameter optimization

AGC target / Compound	2e4		5e4		1e5		2e5		5e5	
	mean	RSD (%)	mean	RSD (%)	mean	RSD (%)	mean	RSD (%)	mean	RSD (%)
Squalene	4.08E+06	8.68	5.44E+06	3.79	8.85E+06	13.17	5.44E+06	6.99	1.94E+06	8.73
Lanosterol	1.02E+07	8.27	4.95E+06	14.27	7.13E+06	4.77	2.48E+06	3.14	9.39E+05	11.23
7 Dehydrocholesterol	3.29E+06	8.78	3.59E+06	13.36	4.96E+06	5.42	4.82E+06	4.07	2.24E+06	4.15
24 Hydroxycholesterol	0.00E+00	0.00	0.00E+00	0.00	0.00E+00	0.00	6.18E+05	6.10	5.09E+04	9.63
25 Hydroxycholesterol	0.00E+00	0.00	0.00E+00	0.00	0.00E+00	0.00	0.00E+00	0.00	0.00E+00	0.00
27 Hydroxycholesterol	0.00E+00	0.00	0.00E+00	0.00	0.00E+00	0.00	0.00E+00	0.00	0.00E+00	0.00
Progesterone	2.27E+07	9.00	2.57E+07	10.70	2.40E+07	12.69	1.81E+07	14.41	1.30E+07	6.11
Corticosterone	1.13E+07	5.33	1.22E+07	7.42	1.12E+07	0.51	1.08E+07	2.99	8.37E+06	7.91
Testosterone	2.38E+07	2.57	2.36E+07	4.66	3.23E+07	6.83	2.92E+07	2.25	1.54E+07	3.25

Table S3-2 AGC Parameter optimization

AGC target / Compound	1e6		3e6		5e6	
	mean	RSD (%)	mean	RSD (%)	mean	RSD (%)
Squalene	1.27E+06	6.76	2.76E+06	7.30	7.65E+05	8.49
Lanosterol	7.40E+05	9.51	2.23E+06	8.63	6.15E+05	3.73
7 Dehydrocholesterol	1.76E+06	12.38	2.83E+06	12.58	1.25E+06	3.96
24 Hydroxycholesterol	3.41E+04	6.80	1.66E+05	11.73	2.16E+04	10.03
25 Hydroxycholesterol	0.00E+00	0.00	2.20E+04	9.68	8.39E+03	9.16
27 Hydroxycholesterol	0.00E+00	0.00	2.39E+04	5.39	9.18E+03	7.77
Progesterone	8.91E+06	3.66	2.49E+06	13.67	5.24E+06	12.82
Corticosterone	6.76E+06	1.66	5.85E+06	4.21	4.15E+06	3.98
Testosterone	1.20E+07	0.48	1.56E+07	8.52	6.52E+06	3.17

Table S4 Injection time optimization

Injection time<br>Compound	100 ms		150 ms		200 ms		250 ms	
	mean	RSD (%)	mean	RSD (%)	mean	RSD (%)	mean	RSD (%)
Squalene	1.16E+06	0.00	3.05E+05	7.46	8.46E+06	5.61	1.19E+06	7.54
Lanosterol	1.08E+06	4.12	9.88E+05	4.88	7.13E+06	2.34	9.93E+05	8.39
7 Dehydrocholesterol	1.45E+05	10.48	1.20E+05	2.70	4.96E+06	7.57	1.20E+05	6.08
24 Hydroxycholesterol	7.90E+03	7.53	1.21E+04	9.06	1.66E+05	5.10	8.10E+03	8.07
25 Hydroxycholesterol	6.31E+03	7.38	7.90E+03	10.02	2.20E+04	1.38	6.23E+03	8.55
27 Hydroxycholesterol	2.14E+04	9.81	2.08E+04	9.21	2.39E+04	2.33	2.08E+04	1.73
Progesterone	1.18E+05	8.20	1.17E+05	4.41	2.40E+07	1.44	0.00E+00	8.21
Corticosterone	4.88E+05	4.58	5.45E+05	5.77	1.12E+07	2.50	4.75E+05	11.29
Testosterone	2.87E+05	9.82	3.19E+05	2.40	3.23E+07	6.32	2.85E+05	7.01

Table S5 Flowrate optimization

Flowrate<br>Compound	50 ul/min		100 ul/min		150 ul/min		200 ul/min	
	mean	RSD (%)	mean	RSD (%)	mean	RSD (%)	mean	RSD (%)
Squalene	0.00E+00	0.00	5.21E+06	2.39	4.31E+05	8.94	1.06E+05	3.21
Lanosterol	5.70E+05	3.21	7.13E+06	3.64	6.76E+05	4.13	0.00E+00	0.00
7 Dehydrocholesterol	0.00E+00	0.00	4.96E+06	4.52	1.05E+08	2.42	1.77E+07	13.75
24 Hydroxycholesterol	7.40E+04	6.61	1.15E+04	2.80	5.57E+04	1.18	1.01E+04	11.30
25 Hydroxycholesterol	1.22E+05	5.02	1.22E+04	9.23	6.04E+03	7.01	1.26E+04	12.34
27 Hydroxycholesterol	2.06E+05	7.99	2.21E+04	7.37	3.48E+04	9.72	3.11E+04	8.30
Progesterone	0.00E+00	0.00	2.40E+07	4.50	2.65E+07	8.33	2.56E+07	13.02
Corticosterone	0.00E+00	0.00	1.12E+04	8.32	9.93E+06	4.82	7.95E+05	10.52
Testosterone	0.00E+00	0.00	3.23E+07	1.23	2.07E+07	4.56	1.55E+07	4.16

Table S6 Needle position optimization

Needle position / Compound	A		B		C		D	
	mean	RSD (%)	mean	RSD (%)	mean	RSD (%)	mean	RSD (%)
Squalene	3.58E+06	7.66	5.48E+06	2.37	4.31E+05	4.29	1.72E+06	9.71
Lanosterol	3.02E+06	1.02	2.47E+06	4.58	6.76E+05	7.37	5.11E+06	8.66
7 Dehydrocholesterol	5.52E+07	12.09	4.89E+07	8.66	1.05E+08	4.14	1.41E+08	11.26
24 Hydroxycholesterol	3.13E+04	9.79	6.91E+04	4.50	5.57E+04	7.58	1.14E+04	13.25
25 Hydroxycholesterol	1.20E+04	4.98	1.09E+04	7.32	6.04E+03	13.70	1.65E+04	8.18
27 Hydroxycholesterol	2.49E+04	10.90	3.09E+04	8.11	3.48E+04	13.89	5.27E+04	5.42
Progesterone	7.00E+06	3.16	1.50E+07	9.23	2.65E+07	8.67	4.39E+07	4.55
Corticosterone	1.45E+07	11.67	1.09E+07	2.42	9.93E+06	6.91	1.73E+07	3.62
Testosterone	3.01E+07	2.09	2.10E+07	7.51	2.07E+07	6.64	2.77E+07	3.28

Table S7 Extraction method optimization

Methods / Compound	Bligh and Dyer		MeOH		SPE	
	mean	RSD (%)	mean	RSD (%)	mean	RSD (%)
Squalene	9.29E+05	4.20	2.22E+06	4.68	1.45E+06	2.36
Lanosterol	2.66E+06	1.12	2.66E+05	7.95	4.10E+05	10.30
7 Dehydrocholesterol	9.84E+04	2.35	1.68E+04	5.48	2.69E+05	7.36
24 Hydroxycholesterol	9.30E+03	4.65	1.68E+04	9.85	3.22E+04	8.35
25 Hydroxycholesterol	1.87E+04	7.63	6.39E+03	5.95	3.22E+04	2.56
27 Hydroxycholesterol	1.38E+04	8.87	1.87E+04	6.34	3.22E+04	6.35
Progesterone	4.27E+04	4.36	5.49E+04	2.35	8.02E+04	9.23
Corticosterone	4.30E+05	4.28	5.74E+05	1.93	2.35E+05	7.33
Testosterone	2.68E+05	1.35	2.02E+05	1.80	6.54E+06	4.89

Table S8 Accuracy and estimated concentration of steroids in killifish tissues

Compound	elemental composition	Δppm	Estimated concentration in tissues young/old (pg/mL)			
			gut	liver	brain	gonad
Squalene	$C_{30}H_{50}$	-1.407	≈0.36-0.33	≈0.35-0.17	≈0.34-0.15	≈0.9-0.45
Lanosterol	$C_{30}H_{50}O$	-2.322	≈21-22	≈21-12	≈6.3-1.6	≈56-32
7 Dehydrocholesterol	$C_{27}H_{44}O$	-0.781	≈20-21	≈12-6	≈25-23	≈42-49
24 Hydroxycholesterol	$C_{27}H_{46}O_2$	-3.938	≈10-9	≈12-10	≈10-5	≈10-13
25 Hydroxycholesterol	$C_{27}H_{46}O_2$	-2.198	≈4-5	≈5-10	≈10-20	≈5-37
27 Hydroxycholesterol	$C_{27}H_{46}O_2$	-2.274	≈2-4	≈0.2-2	≈2-10	≈10-41
Progesterone	$C_{21}H_{30}O_2$	1.634	≈172-50	≈44-40	≈50-23	≈181-40
Corticosterone	$C_{21}H_{30}O_4$	2.648	≈39-44	≈20-25	≈20-25	≈81-62
Testosterone	$C_{19}H_{28}O_2$	-2.919	≈670-698	≈374-113	≈117-112	≈3240-328

Table S9 LC-Gradient

Time (min)	Eluent %B
0.3	10
8	99
10	99
11	10
12	10

Figure S1
Gut Tissue

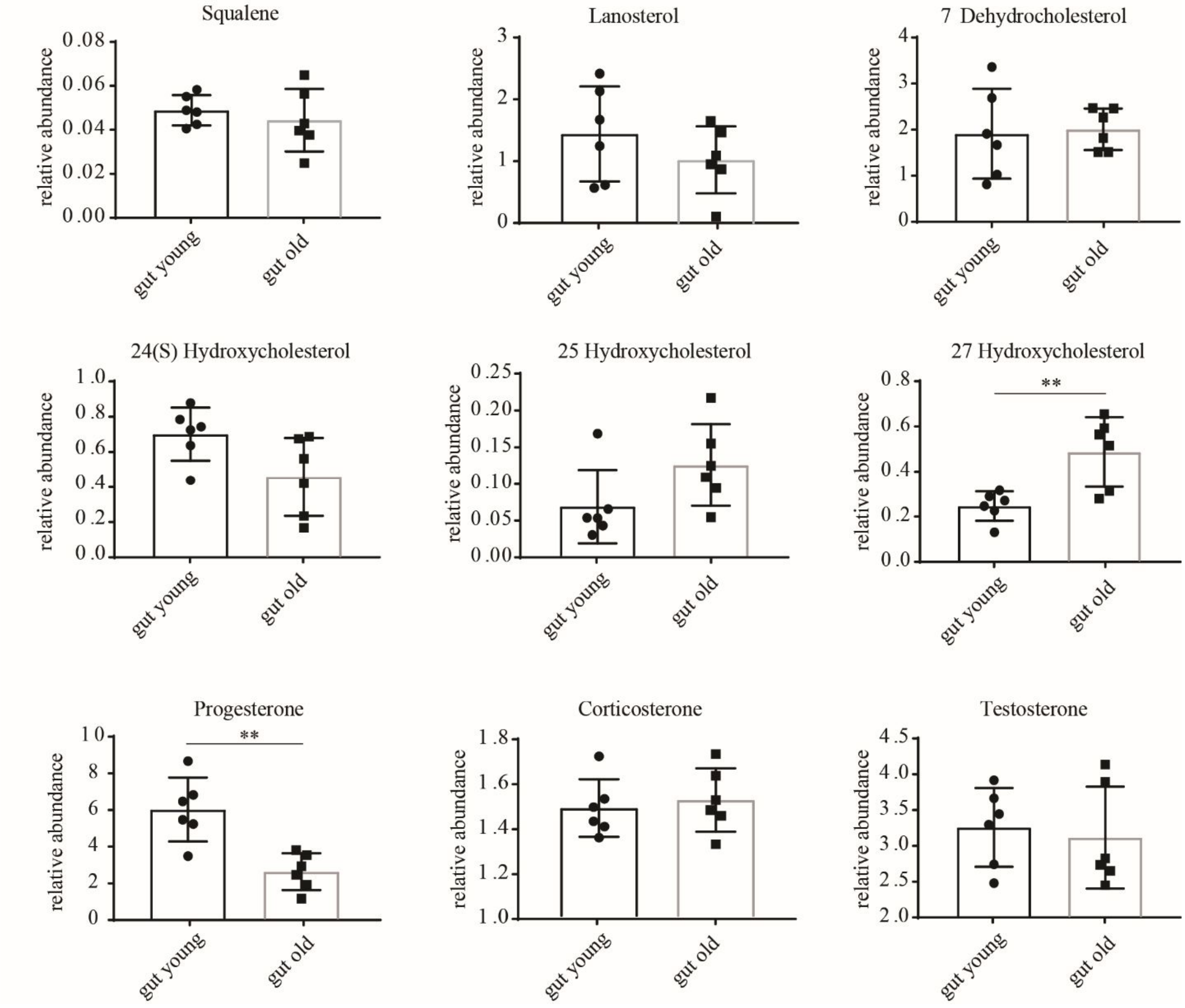

Fig. S1 Quantification of steroid in gut tissue of young and old killifish. Statistical test were performed using t-test. * $P \leq 0.05$, ** $P \leq 0.01$, *** $P \leq 0.001$

Figure S2
Liver Tissue

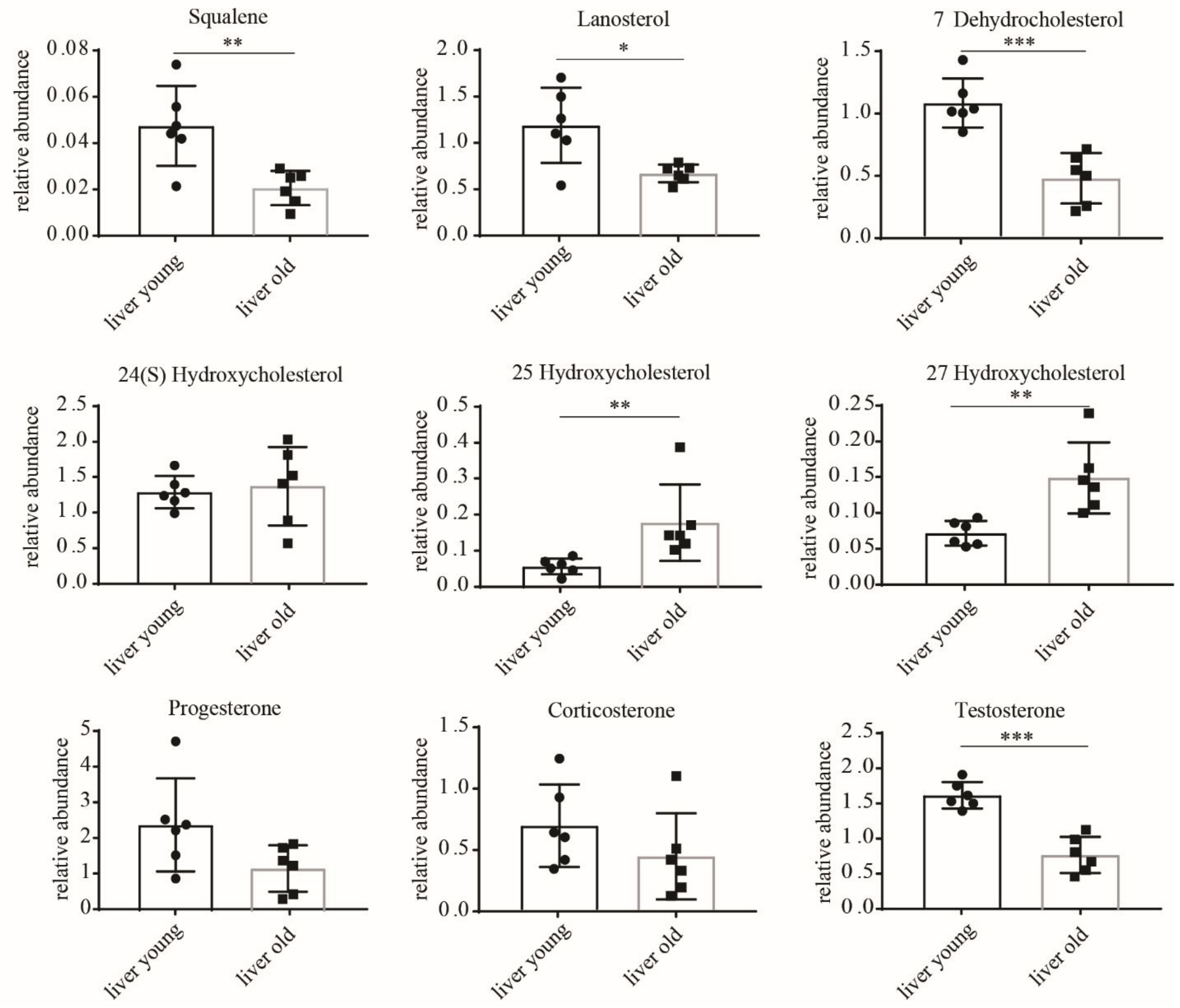

Fig. S2 Quantification of steroid in liver tissue of young and old killifish. Statistical test were performed using t-test. * P ≤ 0.05, ** P ≤ 0.01, *** P ≤ 0.001

Figure S3
Brain Tissue

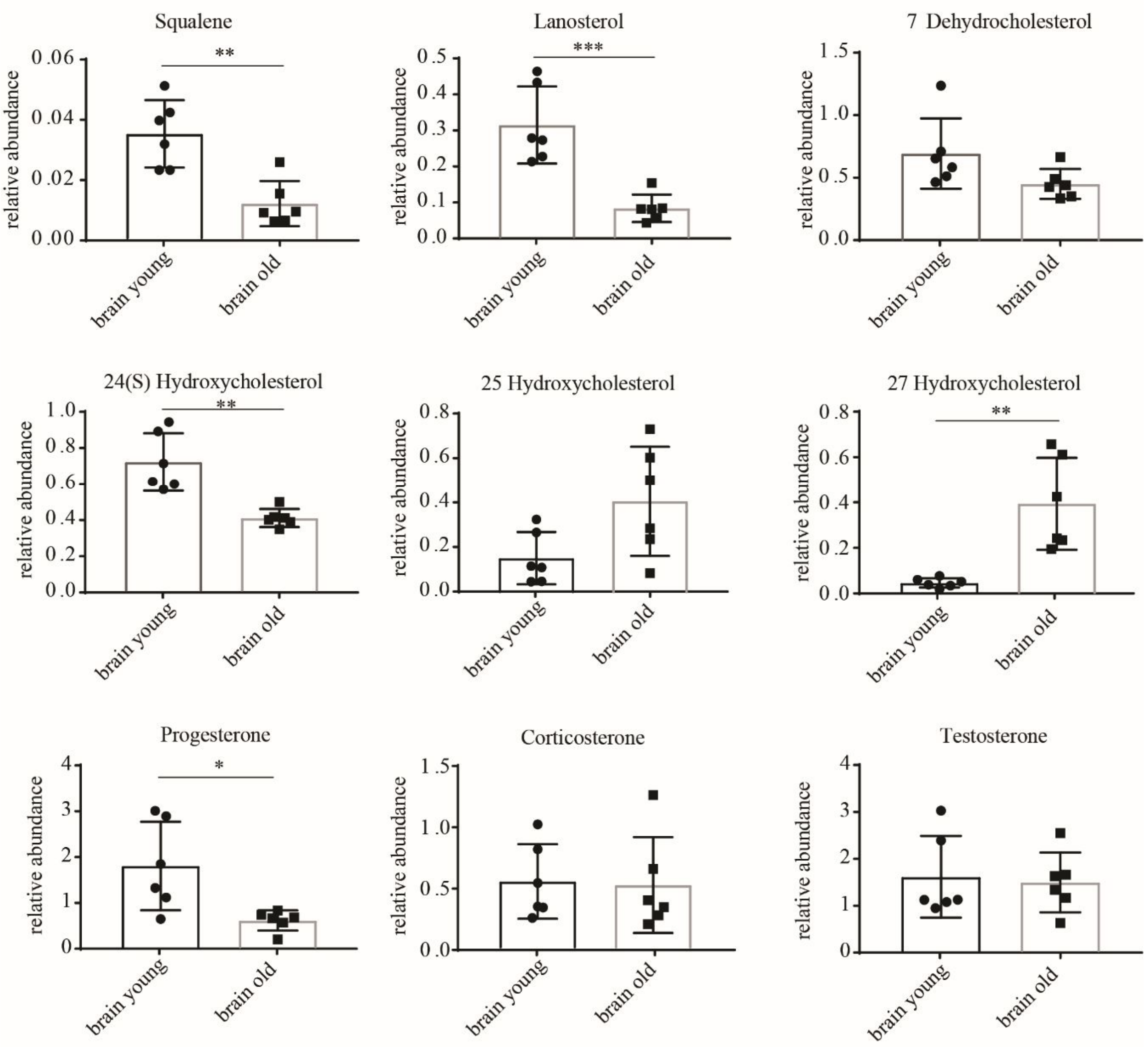

Fig. S3 Quantification of steroid in brain tissue of young and old killifish. Statistical test were performed using t-test. * $P \leq 0.05$, ** $P \leq 0.01$, *** $P \leq 0.001$

Figure S4
Gonad Tissue

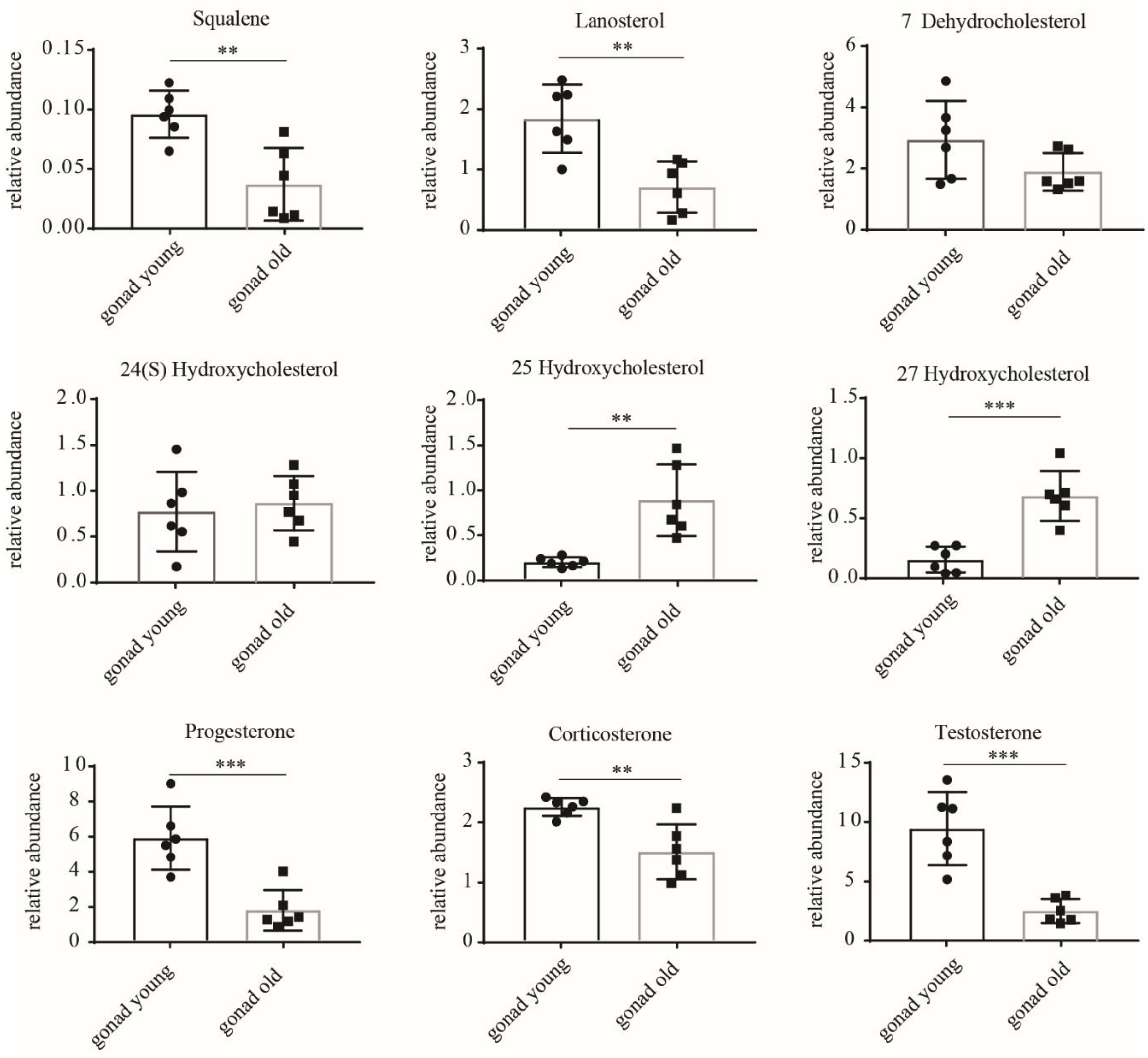

Fig. S4 Quantification of steroid in gonad tissue of young and old killifish. Statistical test were performed using t-test. * $P \leq 0.05$, ** $P \leq 0.01$, *** $P \leq 0.001$

Table S10 Comparison with existing LC-MS based method

Compound	LOQ	Ionisation/Setup	References
Cortisol	2.5nmol/L	HPLC-ESI-MS/MS	(1)
17OH pregnelone	43 pg/mL	HPLC-ESI-MS/MS	(2)
Testosterone	22 pg/mL	HPLC-ESI-MS/MS	(2)
Pregnelone	250 pg/mL	HPLC-ESI-MS/MS	(3)
Testosterone	72 pg/mL	HPLC-ESI-MS/MS	(4)
Testosterone	20 pg/mL	HPLC-ESI-MS/MS	(5)
Testosterone	20 pg/mL	HPLC-ESI-MS/MS	(6)
Testosterone	20 pg/mL	HPLC-ESI-MS/MS	(7)
Testosterone	50 pg/mL	HPLC-ESI-MS/MS	(8)
Lanosterol	50 ng/mL	APCI	(9)
Squalene	50 µg/mL	GC-MS	(10)
Lanosterol	20 µg/mL	HPLC-ESI-MS/MS	(10)
7 Dehydrocholesterol	200 µg/mL	HPLC-ESI-MS/MS	(10)
7-α/β-OHC,	0.5 ng/ml	HPLC-ESI-MS/MS	(10)
25-hydroxycholesterol	4.0 ng/mL	HPLC-ESI-MS/MS	(11)
27-hydroxycholesterol	5 ng/mL	HPLC-ESI-MS/MS	(11)
27-hydroxycholesterol	4 ng/mL	HPLC-ESI-MS/MS	(11)
7-dehydrocholesterol	26.0 ng/mL	HPLC-ESI-MS/MS	(11)
Several sterols	0.01 to 20 ng/mL	HPLC-ESI-MS/MS	(12)

References

1. Wear JE, Owen LJ, Duxbury K, Keevil BG. A simplified method for the measurement of urinary free cortisol using LC-MS/MS. J Chromatogr B Analyt Technol Biomed Life Sci. 2007;858(1-2):27-31. Epub 2007/08/19.
2. Keski-Rahkonen P, Huhtinen K, Poutanen M, Auriola S. Fast and sensitive liquid chromatography-mass spectrometry assay for seven androgenic and progestagenic steroids in human serum. J Steroid Biochem Mol Biol. 2011;127(3-5):396-404. Epub 2011/06/21.
3. Kushnir MM, Rockwood AL, Roberts WL, Pattison EG, Owen WE, Bunker AM, Meikle AW. Development and performance evaluation of a tandem mass spectrometry assay for 4 adrenal steroids. Clin Chem. 2006;52(8):1559-67. Epub 2006/06/17.
4. Haring R, Hannemann A, John U, Radke D, Nauck M, Wallaschofski H, Owen L, Adaway J, Keevil BG, Brabant G. Age-specific reference ranges for serum testosterone and androstenedione concentrations in women measured by liquid chromatography-tandem mass spectrometry. J Clin Endocrinol Metab. 2012;97(2):408-15. Epub 2011/12/14.
5. McNamara KM, Harwood DT, Simanainen U, Walters KA, Jimenez M, Handelsman DJ. Measurement of sex steroids in murine blood and reproductive tissues by liquid chromatography-tandem mass spectrometry. J Steroid Biochem Mol Biol. 2010;121(3-5):611-8. Epub 2010/02/11.
6. Rothman MS, Carlson NE, Xu M, Wang C, Swerdloff R, Lee P, Goh VH, Ridgway EC, Wierman ME. Reexamination of testosterone, dihydrotestosterone, estradiol and estrone levels across the menstrual cycle and in postmenopausal women measured by liquid chromatography-tandem mass spectrometry. Steroids. 2011;76(1-2):177-82. Epub 2010/11/13.
7. Fanelli F, Belluomo I, Di Lallo VD, Cuomo G, De Iasio R, Baccini M, Casadio E, Casetta B, Vicennati V, Gambineri A, Grossi G, Pasquali R, Pagotto U. Serum steroid profiling by isotopic dilution-liquid chromatography-mass spectrometry: comparison with current immunoassays and reference intervals in healthy adults. Steroids. 2011;76(3):244-53. Epub 2010/11/30.
8. Saeves I, Vethe NT, Bergan S. Quantification of 6 glucocorticoids in human plasma by liquid chromatography tandem mass spectrometry: method development, validation, and assessment of matrix effects. Ther Drug Monit. 2011;33(4):402-10. Epub 2011/07/12.
9. Trosken ER, Straube E, Lutz WK, Volkel W, Patten C. Quantitation of lanosterol and its major metabolite FF-MAS in an inhibition assay of CYP51 by azoles with atmospheric pressure photoionization based LC-MS/MS. J Am Soc Mass Spectrom. 2004;15(8):1216-21. Epub 2004/07/28.
10. Muller C, Junker J, Bracher F, Giera M. A gas chromatography-mass spectrometry-based whole-cell screening assay for target identification in distal cholesterol biosynthesis. Nat Protoc. 2019;14(8):2546-70. Epub 2019/07/26.
11. Narayanaswamy R, Iyer V, Khare P, Bodziak ML, Badgett D, Zivadinov R, Weinstock-Guttman B, Rideout TC, Ramanathan M, Browne RW. Simultaneous determination of oxysterols, cholesterol and 25-hydroxy-vitamin D3 in human plasma by LC-UV-MS. PLoS One. 2015;10(4):e0123771. Epub 2015/04/16.
12. du Toit T, Stander MA, Swart AC. A high-throughput UPC(2)-MS/MS method for the separation and quantification of C19 and C21 steroids and their C11-oxy steroid metabolites in the classical, alternative, backdoor and 11OHA4 steroid pathways. J Chromatogr B Analyt Technol Biomed Life Sci. 2018;1080:71-81. Epub 2018/02/27.
